# Time Course of Cultural Differences in Spatial Frequency Use for Face Identification

**DOI:** 10.1038/s41598-018-19971-1

**Published:** 2018-01-29

**Authors:** Amanda Estéphan, Daniel Fiset, Camille Saumure, Marie-Pier Plouffe-Demers, Ye Zhang, Dan Sun, Caroline Blais

**Affiliations:** 1 Département de psychoéducation et de psychologie, Université du Québec en Outaouais, Québec, Canada; 2 Département de psychologie, Université du Québec à Montréal, Québec, Canada; 3 Institute of Psychological Science, Hangzhou Normal University, Hangzhou, China; 4 Zhejiang Key Laboratory for Research in Assessment of Cognitive Impairments, Hangzhou, China

## Abstract

Several previous studies of eye movements have put forward that, during face recognition, Easterners spread their attention across a greater part of their visual field than Westerners. Recently, we found that culture’s effect on the perception of faces reaches mechanisms deeper than eye movements, therefore affecting the very nature of information sampled by the visual system: that is, Westerners globally rely more than Easterners on fine-grained visual information (i.e. high spatial frequencies; SFs), whereas Easterners rely more on coarse-grained visual information (i.e. low SFs). These findings suggest that culture influences basic visual processes; however, the temporal onset and dynamics of these culture-specific perceptual differences are still unknown. Here, we investigate the time course of SF use in Western Caucasian (Canadian) and East Asian (Chinese) observers during a face identification task. Firstly, our results confirm that Easterners use relatively lower SFs than Westerners, while the latter use relatively higher SFs. More importantly, our results indicate that these differences arise as early as 34 ms after stimulus onset, and remain stable through time. Our research supports the hypothesis that Westerners and Easterners initially rely on different types of visual information during face processing.

## Introduction

Perception is the process through which sensory information is organized, categorized and interpreted so as to create a meaningful subjective representation of the outside world. In this sense, perception can be viewed as an inferential process through which sensory input is associated to signification: for long, it has been a widely accepted theory that visual perception involves “unconscious inferences” – i.e. automatic assumptions about the way physical stimuli should appear – based on one’s memories, expectations and attention (see e.g. ref.^1^), and generally, that world knowledge is central to how humans process visual information^2^. That said, the way knowledge about the world is acquired can vary from one culture to another, along with the type of implicit rules that lead to meaningful inferences about visual sensation (see e.g. refs.^3,4^). These culturally circumscribed implicit rules are essential to understand how visual information is coded and associated to stored knowledge. Nonetheless, theories on how visual information is represented and processed have, for decades, rested solely on empirical data from Western, educated, industrialized, rich and democratic (WEIRD) societies^5^, and thus, cannot necessarily be generalized to other populations.

As a response, cross-cultural perspectives on human perception have gained increasing interest since the 20th century. For instance, ongoing research has been investigating the influence of culture on the processing of information contained in visual stimuli, and exposed striking differences in the way Western and Eastern cultures allocate their attention across their field of vision^6–9^. As a matter of fact, culture seems to impact visual processes as basic as spatial frequency (SF) information extraction^10^ during face recognition^11^. Although the effects of culture on perception target such elementary mechanisms, it is still quite unclear at what point during the course of visual information processing these differences between Westerners and Easterners arise, and in what way they unfold to create a visual representation of the world. Therefore, the aim of the present study is to measure the time course of cultural differences during the processing of information from facial stimuli. Studying the onset time of these cultural differences will allow us to get a better grasp of the extent to which the effect of culture on visual processes is etched, whereas the investigation into the time course of this effect can enlighten us on how visual information, encoded in SFs, is added up to form a meaningful image of one’s respective cultural environment.

One of the dominant hypotheses in the literature pertaining to these cultural differences in perception argues that Easterners attend more “holistically” to their visual world than Westerners, whereas Westerners attend more “analytically” to their visual world than Easterners^4,6^. Indeed, evidence gathered from various experimental paradigms has led to this hypothesis^6^. First, Easterners achieve lower accuracy rates than Westerners in simple visual tasks where participants are asked to inhibit contextual information^12,13^. For instance, this tendency is observed when participants are asked to judge the absolute orientation of a line placed in a frame that rotates independently (i.e. judge its orientation regardless of the frame’s presence; the Rod-and-Frame test^14^), or draw a line inside a square as to match the absolute size (i.e. its size regardless of the square’s presence) of another line featured in a square that differs in size (i.e. the Framed-Line test). Moreover, during a change-detection task featuring four uniquely colored squares, Easterners are better than Westerners at detecting changes in the periphery, but worse at detecting changes in the central visual field^8^, which corroborates the propensity of Easterners to attend more to the periphery of their visual field than Westerners.

Second, Easterners’ tendency to process the context more than Westerners has also been observed during the processing of more complex visual scenes. For instance, East Asian participants are more influenced, compared to Western Caucasian participants, by the expression of surrounding faces when asked to judge the intensity of a central figure’s facial expression^7,15^, and have more difficulty than Westerners recalling whether they previously viewed an object if it is presented to them on a different background than the one on which they had first seen it^16,17^. These findings have supported the assumption that Easterners integrate the focal object along with its background during memory encoding, whereas Westerners encode the focal object independently from its background.

Cross-cultural research on the visual processing of faces has unfolded in a way that ties in with the observed cultural differences in attention distribution. Specifically, during learning and recognition, Easterners allocate less fixations to the eyes and mouth than Westerners, and instead direct more fixations near the center of a face than the latter group^18^. Many studies have henceforth replicated these findings using faces as well as other homogeneous object categories – i.e. classes of stimuli that possess a generally uniform shape^19–23^. Some data also highlights that Easterners’ aforementioned eye movement pattern persists despite both cultures attending to and using the same facial areas – the eyes and mouth – to recognize faces^19^. This finding was interpreted as support to the idea that Easterners’ tendency to fixate more than Westerners the central area of a face reflects a greater attentional diffusion to reach the facial areas required to perform the task. Consequently, Easterners’ ability to process the important facial features in peripheral vision might explain why they spend less time than Westerners directly fixating those features.

Nevertheless, eye movements are quite slow in comparison with the time needed to recognize a face, and the degree to which one can rely on this measure to make inferences regarding the cognitive processes underlying face recognition is debatable. As a matter of fact, a study by Or, Peterson and Eckstein^24^ has revealed that the initial fixations during face recognition, i.e. those that are actually necessary for face recognition^25^, land on the same facial area for Easterners and Westerners, thus challenging the hypothesis that culture influences the attentional and visual mechanisms underlying face recognition per se. Instead, the authors proposed that the cultural differences observed in the eye fixation patterns reflect cultural norms that take place later during the stimulus processing. However, an important part of the visual information essential to recognition can be acquired through extrafoveal processing, which can happen during the first fixation^26^. In this sense, later eye movements can be relevant to delineate the patterns of covert visual attention initially deployed. Thus, even though Westerners and Easterners direct their first fixations towards identical facial regions during the recognition of a face, the way they originally respectively spread their extrafoveal attention across the face might differ, and be reflected in their subsequent eye movement patterns.

More recently, we have shown that the impact of culture on face processing goes deeper than the differences revealed in eye movement patterns, and can indeed be observed in the nature of the visual information extracted by both cultures, namely the SFs they use^11^. In fact, in light of evidence suggesting that a broader allocation of attention facilitates low SF information processing, while hindering the processing of higher SFs^27–29^, we directly compared the SF tunings of Westerners and Easterners during two face processing tasks and found a clear cultural difference in the use of SFs, to wit that Easterners relied on lower SFs, and Westerners relied on higher SFs to correctly identify a face. These results offer a strong support to the hypothesis that both cultural groups spread their attention differently over a face, and suggest that culture impacts on relatively early visual mechanisms.

Although our previous study highlighted a strong cultural effect on the SFs that are ultimately more useful for facial recognition, this does not enable us to know when and how this cultural effect occurs. Notwithstanding the need for neurological data to further understand when and how lower and higher level perceptual mechanisms interact in the course of visual information processing, analyzing the variations along the time course of SF extraction makes it possible to get a sense of the earliness of the cultural impact on visual information sampling. More specifically, the onset time of the cultural differences can enlighten us on the extent to which the effect of culture on facial perception is engrained, whereas cultural differences in the time course itself can inform us on the way SFs are integrated across cultures during face identification.

With respect to the onset, an immediate cultural effect on SF extraction could suggest the presence of an early, perhaps overreaching, cultural bias for a specific set of SFs, whereas later cultural differences would suggest that both cultures’ visual systems seem to initially operate in a similar way, and that later processes might be involved in the observed cultural differences. In fact, eye movement studies found similar initial gaze positions – near the center of the face – for Westerners and Easterners during face recognition^24,30^, but diverging fixation patterns throughout longer periods of stimulus observation^18–23^, which seems consistent with a later onset of cultural differences. However, as we previously mentioned, eye movements cannot inform us in real-time about the information that is actually used by an observer, but can only do so at a later time, insofar as they follow prior related attentional strategies. Thus, if Westerners and Easterners deploy their attention differently during those first fixations, it is also possible that the onset of the cultural effect on SF sampling would be immediate, despite similar initial gaze positions, and thus precede the observed cultural differences in eye movements. In this sense, measuring the onset time of the effect of culture on SF sampling can contribute to further understand the link between the observed cultural differences in eye movements and SF extraction. Indeed, the current available data is insufficient to clarify whether the type of visual information sampled is determined by the different eye movement patterns or the opposite.

Regarding the time course, on one hand, if Westerners and Easterners initially use the same SFs, and only later exhibit differences in their use of SFs, this could highlight cultural differences in how facial information is integrated. On the other hand, the SF sampling pattern of Easterners may be globally proportional to that of Westerners, but in comparison, consist in the extraction of altogether lower SFs. In this case, the cultural differences should be consistent through time, and highlight similarities in how both cultures integrate information during a face processing task. By specifically investigating the time course of the cultural differences in SF utilization, this study allows us to untangle these possibilities and acquire more accurate knowledge pertaining to the moment at which culture starts shaping the nature of the visual information sampled during facial recognition and the manner in which this phenomenon unfolds.

Evidence that cultural differences emerge quite early during visual processing of a stimulus has been obtained in a few other studies. For instance, the formerly cited study by Boduroglu, Shah, and Nisbett^8^ highlights divergences in how Easterners and Westerners allocate their attention during a change-detection task while the stimulus display time was limited to 150 ms, hence corroborating the hypothesis that, compared to Westerners, Easterners’ attention is dispersed across a broader area of the visual space quite early during the processing of a display. Furthermore, Lao, Vizioli and Caldara^31^ measured Westerners’ and Easterners’ attentional sensitivity to global or local visual characteristics, and tried to pinpoint the onset time of the corresponding neural activity. They found that for Easterners, but not Westerners, greater repetition suppression – a measure of neural adaptation to redundant information – on the attention-sensitive P1 event-related potential (ERP) occurred for repeated information at the global level compared to recurrent local information, as soon as 80 ms after stimulus onset. Thus, this study reveals evidence for an early attentional bias in Easterners toward the global visual features of Navon hierarchical stimuli, as shown by an attention-responsive ERP.

Notwithstanding the foregoing evidence of Easterners’ early attentional bias toward visual information spanning over a wide visual range, as opposed to Westerners, the experimental paradigms used by previous studies were not devised to measure the temporal unfolding of corresponding differences in the use of visual information. In this vein, the method that was used in the present study was specifically implemented to reveal subtle changes in the time course of visual information extraction. This method, thus, makes it possible to reveal with heightened precision how early culture starts to shape the nature of the visual information sampled during the processing of a stimulus. Furthermore, this study is the first to investigate the earliness and time course of cultural differences in SF use during the recognition of faces, which will contribute to bridge the aforementioned gap between eye movements, attention and visual information extraction.

Along these lines, the present study measures the time course of the cultural differences in SF utilization during face identification by applying a SF filtering method specifically designed for this purpose. Our current method is based on the Bubbles method^32^, specifically on its more recent version focused on SF sampling (i.e. SF Bubbles, namely used in Tardif *et al*.^11^, as well as in several previous studies – see refs^33–39^). This category of methods is designed to isolate parts of all the information contained in a visual stimulus, e.g. local image features, SFs, or orientations (see ref.^40^), in order to understand the relative importance of each piece of information for efficient visual processing.

**Figure 1:**
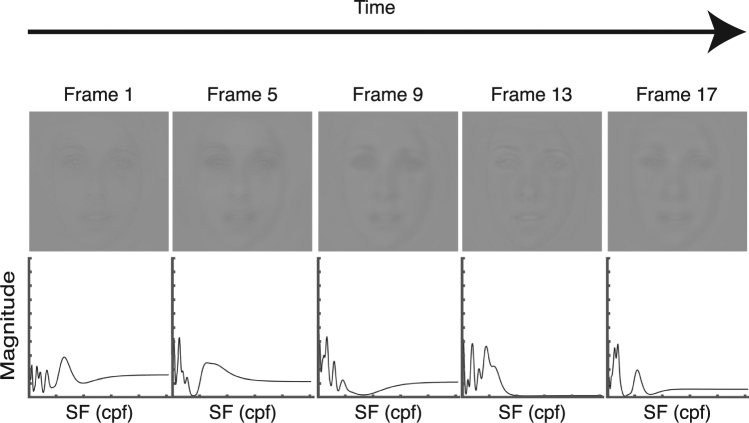
Example of a sequence of the SFs sampled across time, with the resulting image, for one trial. In order to save space, only a subset of the 18 frames were selected for display.

With this in mind, the SF filtering technique used in the current study consists in randomly sampling, on each trial, a subset of the SFs that compose an image – here, faces – and vary this subset through time, within one trial (see Fig. 1 for a stimulus example; see the *Methods* section for more details on the stimuli creation procedure). This allows to measure a participant’s ability to recognize a face containing only the selected SFs at each trial. Since ranges of SFs are more or less likely to be extracted and used by participants at specific time points in the course of visual processing, if the SFs useful for a participant to perform the task are sampled at the right moments, the participant is more likely to respond accurately, and if they are not, the participant is less likely to respond accurately. This method not only allows to assess which SFs are useful for face recognition, but will also give insight on the temporal dynamics of the SFs use as well as the onset of the cultural differences in the use of SFs during the processing of faces.

## Results

### Behavioral performance

First, participants’ accuracy level was entered into a mixed ANOVA with the between-subject factor participants’ culture and within-subject factor stimulus ethnicity. Overall, Canadian participants reached a higher accuracy level (M = 70.29%; SD = 5.57%) than Chinese participants (M = 58.71%, SD = 7.67%), [F(1,43) = 32.634; *p* < 0.001; ${\eta }_{{\rm{p}}}^{2}$ = 0.431]. No significant effect of face ethnicity was found [F(1,43) = 0.859; *p* = 0.359; ${\eta }_{{\rm{p}}}^{2}$ = 0.020], indicating that the stimuli were of comparable difficulty across face ethnicity. However, a significant interaction between participants’ culture and the stimuli’s ethnicity was observed [F(1,43) = 8.707; *p* = 0.005; ${\eta }_{{\rm{p}}}^{2}$ = 0.168]. *Post hoc* t-tests highlight that Canadian participants obtained a significantly higher average accuracy rate with Western Caucasian faces (M = 72.18%; SD = 6.68%) compared to East Asian faces (M = 68.41%; SD = 6.01%), [t(21) = 3.259; *p* = 0.004; Cohen’s d = 0.593; 95% CI: 1.36%, 6.17%], whereas Chinese participants obtained similar average accuracies (M = 57.73%; SD = 9.02% for Western Caucasian faces, M = 59.69%; SD = 7.99% for East Asian faces) across face ethnicities [t(22) = −1.272; *p* = 0.216; Cohen’s d = 0.230; 95% CI: −5.17%, 1.24%]. An equal performance for Chinese participants for both face ethnicities was also obtained in Tardif *et al*.^11^. Interestingly, other studies have also revealed a greater same-race bias for Western Caucasian observers compared to East Asian observers (e.g. see refs^41–43^). It is also important to note that the identities chosen for each face ethnicity category were objectively equalized in recognition difficulty prior to testing, using an ideal observer.

In light of the significant difference in accuracy rates between Canadian and Chinese participants, another mixed ANOVA with the same between and within-subject factors was conducted to verify if there was any difference in the time participants spent practicing with unaltered faces before reaching the main testing phase, during which the dynamic SF filtering was then applied. The analysis revealed no significant main effect of participants’ culture [F(1,40) = 1.299; *p* = 0.261; ${\eta }_{{\rm{p}}}^{2}$ = 0.031] or stimulus ethnicity [F(1,40) = 3.246; *p* = 0.079; ${\eta }_{{\rm{p}}}^{2}$ = 0.075] on the number of practice blocks participants needed to achieve threshold performance in order to start the main task, i.e. a 92% accuracy rate for at least two consecutive blocks with both stimulus ethnicities (Canadian participants: M = 1.11 blocks; SD = 0.32 blocks for Western Caucasian faces, M = 1.58 blocks; SD = 0.96 blocks for East Asian faces; Chinese participants: M = 1.48 blocks; SD = 0.85 blocks for Western Caucasian faces, M = 1.78 blocks; SD = 1.62 blocks for East Asian faces). The interaction between the two factors did not reach significance either [F(1,40) = 0.154; *p* = 0.697; ${\eta }_{{\rm{p}}}^{2}$ = 0.004].

The number of bubbles was also analyzed because, as described in the *Methods* section, it was adjusted at different points throughout the experiment to maintain the accuracy close to a predetermined criterion level; it therefore indexes the amount of information needed, on each trial, by participants to perform the task. Firstly, we focused on the number of bubbles used during the main (third) task. That number was set equally for both face ethnicities based on the final number of bubbles participants reached with other-race faces during the second task. As is mentioned in the *Procedure* section, the final number of bubbles rested on the results with other-race faces because participants were generally worse with those faces. This decision was meant to avoid participants performing at the level of chance with other-race faces. Here, we wanted to verify if the number of bubbles was comparable for Canadian and Chinese participants during the main face identification task, in order to ascertain that this factor did not affect the cultural differences in SF use we might find. An independent sample t-test revealed no significant difference between the average number of bubbles that was used in the main task for Canadian (M = 642.031; SD = 181.269) and Chinese (M = 620.037; SD = 178.680) participants [t(43) = 0.410; *p* = 0.684; Cohen’s d = 0.122; 95% CI: −86.229, 130.217].

In addition, we entered the number of bubbles that participants needed throughout the second task into a mixed ANOVA with the between-subject factor participants’ culture and within-subject factor stimulus ethnicity. This allowed us to measured the average number of bubbles participants initially needed with each face ethnicity to reach the performance threshold, before a stable number of bubbles to use in the main task, was determined. No significant main effect of participants’ culture was revealed [F(1,43) = 0.635; *p* = 0.430; ${\eta }_{{\rm{p}}}^{2}$ = 0.015], albeit a main effect of stimulus ethnicity was significant [F(1,43) = 5.689; *p* = 0.022; ${\eta }_{{\rm{p}}}^{2}$ = 0.117]. However, a significant interaction between participants’ culture and the ethnicity of the stimuli was also revealed [F(1,43) = 15.760; *p* < 0.001; ${\eta }_{{\rm{p}}}^{2}$ = 0.268]. *Post hoc* t-tests indicate that Canadian participants needed significantly more information to recognize East Asian faces (M = 716.581; SD = 169.432), than to recognize Western Caucasian faces (M = 600.893; SD = 169.045) [t(21) = −3.829; *p* = 0.001; Cohen’s d = 0.684; 95% CI: −178.522, −52.854], but Chinese participants require a statistically similar amount of information to recognize both East Asian (M = 605.574; SD = 180.726) and Western Caucasian faces (M = 634.422; SD = 176.332) [t(22) = 1.386; *p* = 0.180; Cohen’s d = 0.162; 95% CI: −14.314, 72.011].

### Spatial frequency × time classification images

The time course of SF utilization was analyzed by producing classification images; they indicate how strongly the participant’s utilization of each SF at each time frame is associated with accuracy (see the *Methods* section for more details on the computation of the classification images). Classification images representing the time course of SF utilization were produced separately for East Asian and Western Caucasian faces. Group classification images representing the time course of SF utilization without regard to the stimulus ethnicity were also produced. Note that analyzing group classification images rather than individual ones is a common practice when using Bubbles (see e.g. refs^33,44–46^); it is one way of reducing the noise inherent to random sampling procedures, as it increases the overall number of trials included in the analysis. Indeed, to be able to identify a meaningful pattern with this method, whether it supports or not the testing hypothesis, a large number of trials is needed to discover a signal (i.e. some regularity) through the trial-by-trial random combinations. Alternatively, it is possible to compute meaningful individual classification images if each individual performs a very large number of trials. This can be useful if individual differences are expected. By comparison, group classification images are typically performed when individuals from that group are expected to be part of the same population, which is the case in the present study on cultural group differences.

**Figure 2:**
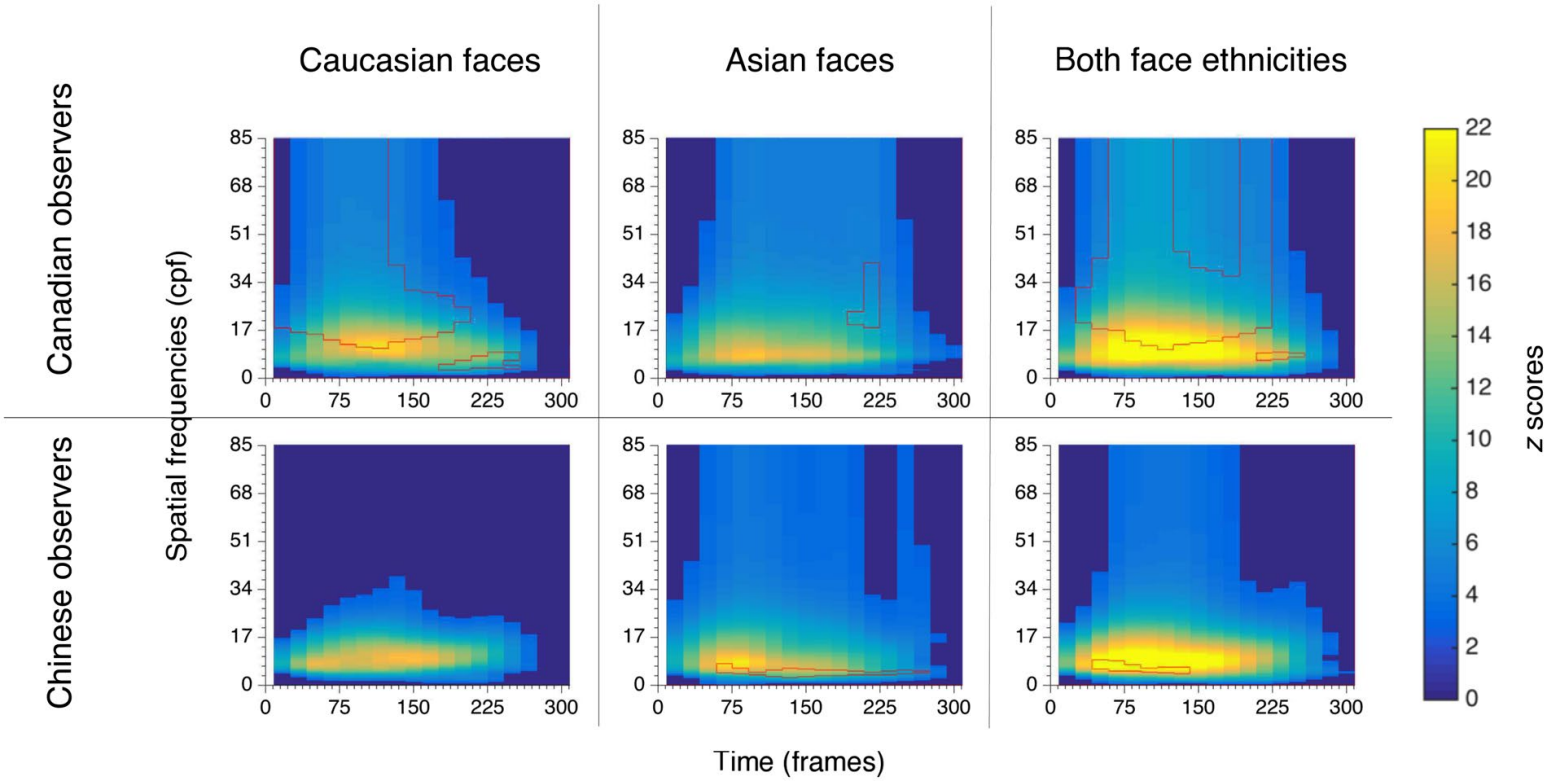
Classification images illustrating Canadian and Chinese observers’ significant use of spatial frequencies across time, for Western Caucasian faces, East Asian faces and both face ethnicities combined. Group differences (i.e. Canadian observers - Chinese observers) are marked for each group and stimulus category: red edges delineate significant SF use biases for each cultural group.

To reveal the SF × time information extraction that was significantly associated with accuracy, the Cluster test from the Stat4CI toolbox (*p* < 0.05; FWHM = 4.47; *Z*crit = 3.0)^47^ was applied to each cultural group’s classification images. This test is based on the random fields theory and corrects for multiple comparisons (i.e. one test per SF and time frame) by controlling for the family-wise error rate, while taking into account the fact that contiguous SFs and time frames are not independent (i.e. at a functional level). The significant clusters are represented in colours other than dark blue on Fig. 2. To uncover statistically significant cultural differences, Chinese group classification images were subtracted from Canadian group classification images, and Cluster tests were applied to these differential classification images (*p* < 0.025; FWHM = 4.47; *Z*crit = 3.0). The clusters representing significant cultural differences are delineated in red on Fig. 2.

The results reveal significant cultural differences akin to those that were found previously^11^. In order to simplify the description of these differences across time, the mean lower and higher bounds of SF utilization were derived for each SF × time cluster; however, the lower and higher bounds of SF utilization on each frame separately are available on Fig. 2. When both face ethnicities are considered together (i.e. Fig. 2, last column), results show that, between 50 and 133 ms of stimulus presentation, Chinese participants benefit significantly more than Canadian participants from lower SFs between 4.8 and 7.3 cycles per face (cpf) to correctly identify faces. In contrast, as early as 34 ms following stimulus onset and for the next 184 ms of stimulus presentation, Canadian participants benefit significantly more than Chinese participants from higher SFs between 16.2 and 60.1 cpf.

Upon analyzing face ethnicity-specific cultural differences (i.e. Fig. 2, first and second columns), the results indicate that Canadian participants make a much greater use than Chinese participants of SFs ranging from 16.7 to 41.5 cpf, as soon as the stimulus appears and for a steady 200 ms, for the identification of Western Caucasian faces. Their high SF bias arises much later and more briefly for East Asian faces (from 18.5 to 32.3 cpf, between 200 and 220 ms), although a trend towards a similar bias can be observed earlier in time; in fact, SFs that fall within the significant cluster revealed for Canadian participants when both face ethnicities are combined (i.e. from 16.2 to 60.1 cpf, between 34 and 217 ms) are also relatively more useful for this group to identify East Asian faces (average *Z* score of 1.48). For Chinese participants, a low SF bias between 3.6 and 5.5 cpf, starting at 67 ms and ending at 267 ms is significantly present for East Asian faces. There was also a trend in the same direction for Western Caucasian faces for SFs that fall within the significant cluster revealed when face ethnicities were combined (i.e. from 4.8 to 7.3 cpf, between 50 and 133 ms; average *Z* score of −2.32). It is also interesting to note that our Canadian participants start making greater use than our Chinese participants of lower SFs ranging from 3.3 to 7.6 cpf later during the course of information extraction (between 184 and 250 ms); this low SF bias is significant only with own-race faces, although there is a trend in the same direction with other-race faces, for SFs that fall within the corresponding cluster of significant low SFs when both face ethnicities were combined (from 6.9 to 9.1 cpf, between 200 and 250 ms; average *Z* score of 1.45).

While this does not jeopardize the validity of the main results, it is worth noting that the sampling method makes it difficult to draw conclusions regarding the upper bounds of the SF ranges used by participants. Indeed, the application of a logarithmic SF sampling technique impacts on the resolution that we may expect in the results. More specifically, the higher the sampled SF, the broader the range of surrounding SFs included in the filter. As explained in the *Methods* section, the decision to use a logarithmic sampling stems from our knowledge of the visual system’s relative sensitivity to SFs; that is, retinal cells which are sensitive to high SFs tend to react to a broader range of SFs than cells which are sensitive to low SFs^10^. Thus, although extremely high SFs are included in the significant clusters of information utilization, it is unlikely that they were actually beneficial to the participants. Rather, they likely are an artifact of the lower resolution of the method with high SFs. An analysis aiming at examining in greater detail the relationship between the sampled SF and the resolution is provided as *Supplementary Information*. That analysis suggests that the presence of extremely high SFs in the significant clusters likely reflects that SFs at least as high as 22 cpf were beneficial to the participants. However, because of the lower resolution with high SFs, it is not possible to know the upper bound of the SFs extracted.

In addition, for the purpose of investigating whether or not our results can be found across a majority of individual participants, we conducted a bootstrap analysis in which participants are randomly sampled (with replacement) to form new samples including overall the same number of subject data. This procedure allows to verify if our results can be found across a majority of random samples of participants, in which the data of any individual participant has varying degrees of weight. We resampled our participants 1000 times and verified if the previously revealed cultural group differences could be found in at least 95% of cases. As a matter of fact, our bootstrap analysis confirms that all but one significant cluster revealed in our main analysis were observed with a 95% confidence interval. Indeed, only the low SF cluster previously found for our Chinese participants when both face ethnicities were combined did not reach the 95% threshold. However, a trend was present for 90% of the samples. This highlights, nonetheless, a greater heterogeneity of processing strategies among Chinese participants, especially with other race faces (see detailed results and figures for this analysis in *Supplementary Information*).

### Spatial frequency tuning across time

**Figure 3:**
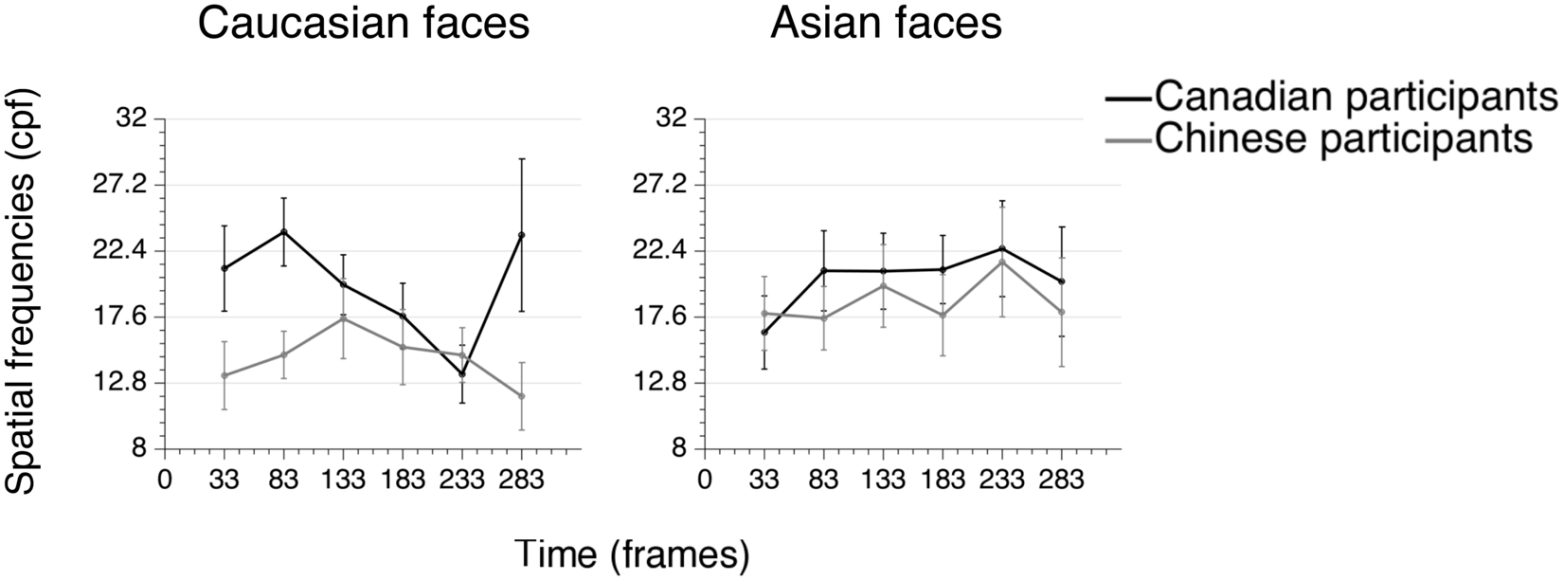
Canadian and Chinese observers’ SF tuning peaks (group average), for Western Caucasian and East Asian faces respectively.

In order to get a better grasp of the time course of SF utilization for both cultures, a second analysis was conducted to verify how the peaks of SF tuning unfolded through time. The participants’ individual SF tuning peaks were calculated at each time-point using the 50% Area Spatial Frequency Measure (ASFM; see ref.^37^). The ASFM method works by finding the SF point that separates the surface underneath the SF tuning curve (i.e the curve representing how strongly each SFs is associated with accuracy) and over the significance threshold in two equal parts. This measure aims to reveal the SF value that approximately characterizes an observer’s SF use preferences, by considering both the highest point (i.e. the largest SF value) and the width (i.e. the SF range) of the curve. As the *Z* score values constituting the individual classification images are overall lower than the values that make up the group classification images, the significance threshold applied here was half of the threshold used for the Cluster test on the group classification images (i.e. *Z*crit = 1.5). For the frames that contained no values that met the threshold, the raw maximum value was used. Furthermore, since the smoothing procedure across time makes it so that a SF sample is present during approximately three time frames (see the *Methods* section, as well as the *Supplementary Information*, for more details), the individual tuning peaks were calculated for six time-points each comprised of an average of three consecutive time frames. This decision also allowed to reduce the number of comparisons needed for the within-subject factor *time frames* of the postliminary analysis of variance. Figure 3 displays the unfolding of SF tuning peaks through time.

Thus, a 2 × 2 × 6 mixed ANOVA, with participants’ culture as a between-subject factor, and face ethnicity and time frames as within-subject factors, was performed on the individual peaks. The main effect of culture was significant [F(1,43) = 4.639; *p* = 0.037; ${\eta }_{{\rm{p}}}^{2}$ = 0.097], meaning that Canadian participants had higher tuning peaks (all frames (cpf): M = 19.9; SD = 15.1 for Western Caucasian faces, M = 20.4; SD = 14.3 for East Asian faces) than Chinese participants (all frames (cpf): M = 14.6; SD = 11.5 for Western Caucasian faces, M = 18.8; SD = 15.2 for East Asian faces). This result replicates the group differences found in the classification images and discussed in the precedent section. It supports the idea that the differences observed in the group classification images reflect the strategy of the whole group rather than the one of just a few unrepresentative individuals. Neither a significant effect of time [F(1,43) = 0.378; *p* = 0.775; ${\eta }_{{\rm{p}}}^{2}$ = 0.009], of face ethnicity [F(1,43) = 1.939; *p* = 0.171; ${\eta }_{{\rm{p}}}^{2}$ = 0.043], nor a significant interaction between factors was found.

### Analyses with accuracy as factor

Finally, in order to rule out the possibility that our results might be driven by the accuracy differences between the two cultural groups, we performed once again all the previous analyses while controlling for participants’ accuracy. This exact procedure was also done in our first study on how culture influences SF use^11^. Firstly, no significant correlation between ASFM peaks and accuracy was found (Canadian participants: r = 0.1; *p* = 0.658, Chinese participants: r = 0.105; *p* = 0.633). The average ASFM peaks across time frames were used here, since our initial analysis on SF tuning peaks did not reveal a significant effect of time.

Then, we matched subgroups of participants on the basis of accuracy rate (12 Canadian participants: M = 66.67%; SD = 4.75%, 12 Chinese participants: M = 64.67%; SD = 3.65%, t (22) = 1.156; *p* = 0.26), chosen according to the same criteria as our previous study^11^: (a) to include as many participants as possible, and (b) to minimize the difference in accuracy rates.

Results on group classification images were similar to the ones found in our initial analysis (results are illustrated in Fig. S3, provided in the *Supplementary Information* file). Overall, between 34 and 150 ms of stimulus presentation, Chinese participants make better use than Canadian participants of SFs between 4.8 and 8.2 cpf. This low SF bias for Chinese participants is present with East Asian faces (from 3.3 and 8.7 cpf; between 34 and 150 ms), whereas, again, only a tendency is observed with Western Caucasian faces for SFs that fall within the significant cluster revealed when face ethnicities were combined (i.e. from 4.8 to 8.2 cpf, between between 34 and 150 ms; average *Z* score of −2.187). By comparison, Canadian participants are better than Chinese participants at recognizing faces with SFs from 19.6 to 54 cpf, between 34 ms and 100 ms of stimulus presentation. This high SF bias for Canadian participants (i.e. from 15.3 cpf and up) occurs as soon as the stimulus appears on screen and remains for 117 ms with Western Caucasian faces, but only during a brief time period with East Asian faces (i.e. between 200 and 217 ms, for SFs from 16.3 to 26 cpf).

Although the mean SF tuning peaks of the cultural subgroups are similar to the ones we obtained with all the participants included, the main effect of culture did not remain significant [F(1,22) = 1.532; *p* = 0.229; ${\eta }_{{\rm{p}}}^{2}$ = 0.065], likely due to the small sample size of our subsamples. Nonetheless, the trend persisted in the same direction [Canadian participants (mean peaks across frames; cpf): M = 18.3; SD = 9.3 for Western Caucasian faces, M = 20.4; SD = 6.9 for East Asian faces; Chinese participants (mean peaks across frames; cpf): M = 14; SD = 7.7 for Western Caucasian faces, M = 18.9; SD = 8.1 for East Asian faces]. In addition, there was still no significant effect of time [F(1,22) = 0.204; *p* = 0.89; ${\eta }_{{\rm{p}}}^{2}$ = 0.009], no significant effect of face ethnicity [F(1,22) = 2.307; *p* = 0.143; ${\eta }_{{\rm{p}}}^{2}$ = 0.095] and no significant interaction between factors.

According to these results, it seems unlikely that the cultural differences revealed earlier in this study could be explained by differences in performance rate.

## Discussion

Respectively influenced by ancient Chinese and Greek civilisations, modern Eastern and Western societies have built diametrically opposite cultures and ways of life^6^. The social structure in which individuals from these respective cultures are brought up heavily shapes their way of gathering knowledge about the visual world, and thus draws their attention to particular aspects of their environment. This systematic attentional bias that arises can influence the very nature of the information sampled by the visual system. Indeed, we have previously shown that culture can shape basic visual processes such as the extraction of SF information during the recognition of a face^11^, which happens to be a powerful social stimulus. The current study went further still and set out to investigate the time course of the cultural differences involved in the effective utilization of SF information for facial recognition. To the best of our knowledge, this research is the first to directly measure the culture-specific time course of SF extraction, by applying an image processing technique specifically designed for this purpose, fitted with empirically proven temporal and spatial accuracy (see *Supplementary Information*).

### Earliness of cultural differences

First and foremost, our findings broadly replicate the differences revealed in our previous study^11^ pertaining to the type of information used by East Asian and Western Caucasian observers during face identification, while using different groups of participants, a different set of stimuli and a modified experimental method and design. That is, Easterners use relatively lower SFs than Westerners, while Westerners have a greater bias for higher SFs. These results corroborate the hypothesis that culture shapes the visual mechanisms underlying face recognition, and that the observed cultural differences in eye movement patterns (see e.g. refs^18,19^) most likely reflect different face recognition processes. Furthermore, although we did not monitor eye movements, the stimulus presentation was constrained to 300 ms. This constraint allows to further our understanding of the link between the culturally divergent eye movement strategies and the utilization of different SFs, namely whether eye movements determine the type of visual information sampled or vice versa. In fact, the short presentation time has likely restricted participants’ eye movements to one or two fixations. Since previous studies have found no cultural difference in the early pattern of fixations of Westerners and Easterners during face recognition^24,30^, our results point to the likelihood that the particular SF extraction strategies used by each cultural group could be influenced by distinct covert attentional patterns rather than eye movements, which in turn guide the culture-specific fixation strategies previously discovered. As a matter of fact, the link between prior covert attention shifts and the deployment of eye movements has been stressed across numerous studies^48–51^.

On a related note, separate studies have shown 1) that Easterners need the same information as Westerners, which is located in the eyes and mouth areas, to recognize a face, but that, unlike Westerners, Easterners use those features without fixating them directly, and 2) that, during that same task, Easterners rely on lower SFs than Westerners. Put together, this data points to the idea that Easterners tend to process the relevant facial features using peripheral vision and, thus, extract the lower SFs located in those features. It would nonetheless be worthwhile, at a later stage, to directly explore the location-specific use of SF information across cultures.

On the other hand, a noteworthy study about cultural differences in facial expression recognition found that Easterners seem to allocate a significantly higher processing weight to the area of the eyes^52^, a pattern that is also highlighted in their mental representations of emotions^53^. Along these lines, considering that Easterners tend to use a more specific region of the face when it come to facial expression recognition, it would be interesting to examine whether this cultural group would also accomplish this task more accurately with lower SFs.

Most importantly, the method used in the present study highlighted that cultural differences in the use of SFs arise quite early during the course of visual information extraction; i.e. as early as 34 ms. Indeed, the method applied was specifically implemented to reveal subtle changes in the time course of visual information extraction, so as to enable us to discover with increased precision the point in time at which culture starts shaping the nature of the visual information sampled during the processing of a stimulus. Although the present study has contributed valuable insights with regard to how early culture impacts on visual information extraction during face processing, a gap still needs to be bridged between the present results and the potential cultural differences in how SF information is processed across the neural visual pathway involved in face processing. Studies integrating a SF sampling technique to ERP data would be useful to pinpoint the visual processing level corresponding to the early cultural differences in SF extraction and use that were laid bare by the current study. In fact, the rapidity at which cultural differences in information extraction occur may reflect early visual processing stages that take place within the early visual cortex and are not specific to faces, i.e. are not face-selective^54,55^. If cultural differences in SF use were indeed shown to be linked to these early processing stages, it would suggest that culture begins to affect perception at a very basic and general processing level of the visual system from which more specific anterior visual processing pathways stem. In fact, evidence from transcranial magnetic stimulation (TMS) suggest that face-selectivity in the occipital face area (OFA) - the earliest visual area solicited for facial information processing^56^ - starts at around 100 ms following stimulus onset, whereas an earlier implication of the OFA at around 50–60 ms is not face-selective^57^. This may furthermore entail that cultural differences in SF sampling could generalize to other categories of stimuli beyond faces, an avenue that should be explored.

### Time course of cultural differences

The classification image analysis indicated that the cultural differences in SF information utilization seem to remain consistent throughout time, for about 220 ms. In further analyzing the time course of the SF tuning peaks we found no significant effect of time, and no interaction between time and cultural group. The present method was designed to allow the detection of changes in the SFs sampled at different moments during visual processing, for instance, as one could have predicted assuming the presence of a cultural influence on the integration of SFs through time. However, despite the relatively good temporal resolution of the method (see *Supplementary Information*), our results do not support variations of the SF utilization through time. For that matter, several studies suggest that SF sampling patterns are flexible, and depend on attentional selectivity and task requirements^58–61^, suggesting that higher level processes may orient the chronology of SF information extraction. On the other hand, in an effort to explain the hypothesis that SF extraction generally follows a coarse-to-fine pattern, Bar^62^ proposed a model that highlights the importance of contextual information for the recognition of natural objects within a complex visual scene and, thus, the role of very early low SF extraction as a contextual canvas that increases subsequent local object recognition efficiency. According to this model, the extraction of higher SFs occurs later in the process to enhance object features for better discrimination, and is facilitated by prior processing of low SFs. Thus, it is possible that the face identification task used in this study did not warrant the need for participants to resort to a systematic coarse-to-fine SF extraction pattern as the featured stimuli were not made up of a complex array of miscellaneous elements, an arrangement that typically characterizes visual scenes. In contrast, using single objects, Caplette, Wicker and Gosselin^63^ recently revealed the use of a coarse-to-fine SF sampling pattern in neurotypical observers (as opposed to those with diagnosed Autism Spectrum Disorder - ASD) during object recognition tasks. Although visually much simpler than scenes, objects, unlike faces, typically have a general heterogeneous shape which increases the likely usefulness of leading coarse-grained, followed by fine-grained, visual information for more efficient recognition. Nevertheless, our results, marked by a temporally stable cultural difference in the use of SFs, seem to support the hypothesis that Westerners and Easterners display an early and steady, culturally specific, SF sampling bias when recognizing a face.

### Other-race effect

Although the aim of the present study was to understand how culture impacts on the time course of SF sampling, the experimental design also allowed to highlight interesting results with respect to the theoretical grounds of the other-race effect. In fact, the results did not indicate an interaction between culture and face ethnicity on the SF tuning of participants, suggesting that individuals do not modify their strategy of SF extraction as a function of the ethnicity of the face processed. The analysis on group classification images supports this finding: although slight differences are observed in the SFs used with each face ethnicity, these differences did not interact with the effect of culture. Indeed, the data points to the conclusion that, for a given culture, similar SFs were correlated with accuracy for both face ethnicities, but that the association was stronger for own-race than other-race faces. The finding that similar SFs are used with both face ethnicities replicates the results we had obtained precedently while using a different set of faces, a different method (SF sampling without the time dimension), and different groups of participants^11^. However, in that previous study, we remained cautious in our interpretation because an Ideal Observer analysis had revealed that the Chinese faces selected were objectively more difficult to discriminate from one another than the Western Caucasian faces, a difference that could have influenced the SFs sampled. In the present study, however, we used an image matching Ideal Observer algorithm prior to the stimulus selection to ensure that faces from both ethnic categories used in the experiment were of comparable difficulty to identify. We can thus now conclude with more confidence that individuals keep using a similar SF sampling strategy when they process same-race as well as other-race faces. This result is also in line with the finding that similar eye movements are observed during the processing of own-race and other-race faces (see e.g. refs^18,19,21^), although others have found otherwise (see e.g. refs^64,65^).

## Conclusion

Over the most recent few years, cross-cultural research trends have been drawing sharpening attention to queries on the nature and extent of the cultural differences observed in the course of various visual tasks. Within this scope, the present article is an valuable contribution to the ongoing inquiry into how and when culture starts to tint the mechanisms and processes involved in visual recognition. This study used a SF filtering technique fine-tuned for temporal and spatial precision to uncover a considerably early cultural effect on the extraction of SF information during face identification, one that potentially falls within the processing time range of early visual areas (i.e. <100 ms^55^). In light of this, a symmetrically fine-tuned investigation into the early neural substrates of visual face and object recognition should examine whether or not culture affects early overreaching visual processes, not circumscribed to specific stimulus categories.

## Methods

### Participants

Twenty-two Western Caucasian Canadian (7 men; mean age of 24; SD = 2.4) and twenty-three East Asian Chinese (7 men; mean age of 21; SD = 1.9) participants completed the task. Chinese participants were tested in Hangzhou (Zhejiang province), were all born in China, lived in China and had little to no experience with occidental cultures. Canadian participants were tested in Gatineau (Quebec province), were born in Canada, lived in Canada, and had little to no experience with oriental cultures. All participants had normal or corrected-to-normal vision. Sample size was based on our previous article regarding cultural differences in SF use, in which a similar method was used^11^, as well as other studies that made use of the SF Bubbles method^33,39^. Given our sample size and an expected F-test effect size (${\eta }_{{\rm{p}}}^{2}$) of 0.188, we have approximately 91% statistical power (as measured by G*Power 3.1) to observe an effect of this size with a repeated-measures mixed ANOVA, as will be applied in the present study. The theoretical effect size was derived from the average effect sizes across all significant two-way or three-way interactions found in several relevant studies, and one meta-analysis, pertaining to either cultural perception, facial or expression recognition (i.e. effect sizes: 0.08^66^, 0.12^67^, 0.14^68^, 0.28^69^, 0.32^68^).

The experimental protocol used in this study was approved by the Institutional Review Boards of Université du Québec en Outaouais and Hangzhou Normal University; all experiments conducted conformed to relevant guidelines and regulations with regard to the use of human participants. Each participant signed a written, informed consent form prior to the experiment.

### Material and stimuli

All tasks were run on the MATLAB software with the Psychophysics Toolbox^70,71^. Western Caucasian face images were drawn from the Radboud^72^, KDEF^73^ and the PICS^74^ databases, and Chinese face images were drawn from the CUFS^75^ and from William Hayward’s face database. All faces displayed a neutral facial expression. Accidental local features such as brown spots or rashes were removed using the Photoshop software. Faces were aligned as well as possible, using as parameter the least-square measure, on the positions of eyes, nose and mouth – by means of translation, rotation, and scaling. They were revealed through a uniform mask to hide external facial features such as hair and ears. Subsequently, the luminance and spatial frequency content were equalized throughout all face images using the SHINE toolbox^76^. Finally, for each face ethnicity, a subset of 8 identities was selected from a pool of 130 faces by using a custom image matching algorithm to ensure that both subsets contained images that were inherently of comparable difficulty to discriminate from an Ideal Observer perspective.

Due to limited material resources, stimuli were displayed on calibrated LCD (52 × 29 cm; 1920 × 1080 p) monitors for the experiment in Canada, and calibrated LCD (37 × 30 cm; 1024 × 768 p) and CRT (32 × 24 cm; 1024 × 768 p) monitors in China. In Canada, the stimuli face had an on-screen width of 5 cm and participants were seated at a viewing distance of 47.7 cm. In China, with the CRT monitors, the stimuli face had an on-screen width of 6 cm and participants were seated at a viewing distance of 57.1 cm, and with the LCD monitors, the stimuli face had an on-screen width of 7 cm and participants were seated at a viewing distance of 66.5 cm. To ensure that viewing distance was constant throughout the entire experiment, all participants were asked to position their head on a chin and forehead rest, facing the screen at the appropriate viewing distance. In consideration of the foregoing, the use of two different types of monitors in no way invalidates our results as the viewing distance was adjusted relative to the on-screen size of the stimuli so that the image face width ultimately subtended 6 degrees of visual angle. This means that the proximal stimuli (retinal images), and thus the actual SFs projected on the retina, were similar in both countries. Furthermore, all monitors had a calibrated luminance and a refresh rate of 60 Hz. In addition, Chinese participants had been tested with both LCD and CRT monitors in our previous study on how culture influences SF use^11^, and the results were not affected by the type of monitors used.

The stimuli were produced in real-time using a transient SF filtering technique. From a conceptual point of view, the method worked as follows. On every trial, one stimulus consisted in a series of faces each representing one ‘time frame’. Each trial was composed of eighteen frames. One by one, all image frames independently underwent a SF filtering process. More specifically, among all the SFs available in the image, a subset was randomly sampled. Thus, each of the eighteen frames represented a face in which different subsets of SFs were available. The eighteen frames were first sampled independently from one another, so the SFs on different time frames varied randomly. However, the eighteen frames in the final stimuli were not completely independent from one another, because a temporal smoothing was applied to avoid abrupt changes in the SFs presented. This temporal smoothing had for effect to create an overlap in the SFs sampled in three successive frames. A spatial smoothing was also applied such that when one SF was sampled, its neighbouring SFs were also sampled. The number of neighbouring SFs that were sampled at the same time as the target SF depended on the specific target SF sampled: the higher it was, the more neighbouring SFs were sampled at the same time. This variation was implemented to adjust the SF selection to the human visual system’s sensitivity to SFs (see ref.^10^). Thus, in the following, what is called a ‘bubble’ is the smooth sampling of the randomly targeted SF along with its SF neighbours.

**Figure 4:**
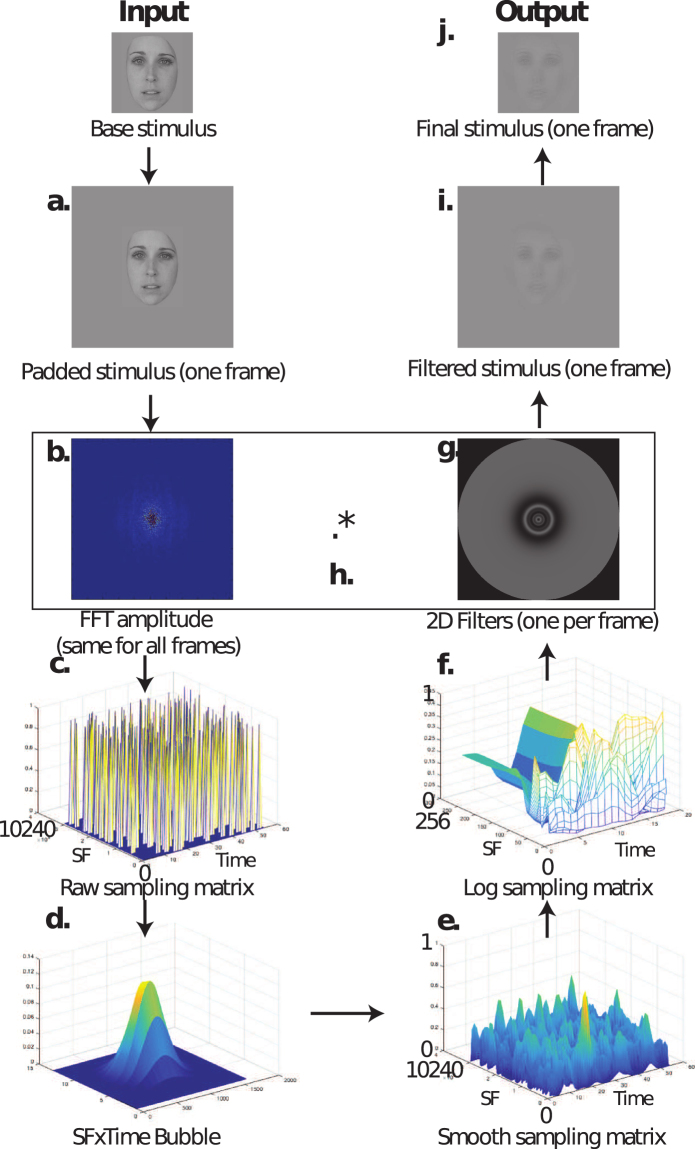
Example of the creation of one stimulus with the temporal SF filtering method.

On a more technical ground, the creation of one stimulus integrates a manifold of computational steps illustrated in Fig. 4. First, each 256 × 256 pixels image frame is centered on a uniform background twice its size as a means of minimizing edge artefacts in the SF domain (Fig. 4a). Second, the padded image is converted to a complex amplitude matrix – representing the SF spectrum of the image – by way of a fast Fourier transform implementation (Fig. 4b). Third, a random matrix of 10,240 × 54 elements containing N ones – representing the number of target SFs sampled – distributed among zeros is generated (raw sampling matrix; Fig. 4c). This raw sampling matrix is the basis to create the eighteen SF filters that are used for each individual image frame. Then, a two-dimensional Gaussian kernel with standard deviations of 1.5 cycles per image (cpi) and 1.3 frames for the dimensions of SF and time respectively is produced, and is convoluted with the raw sampling matrix to create smooth ‘bubbles’ through SFs and time (smooth sampling matrix; Fig. 4d,e). This smoothing procedure makes it so that the SF information sampled at a given frame starts gradually appearing during the previous frames and gradually disappearing during the subsequent frames. The SF information is thus present with at least half of its maximum intensity for about three frames (Full Width at Half Maximum, or FWHM, of 3.06 frames), allowing for a visually smooth transition across time. Fourth, the smooth sampling matrix is re-sampled along the dimension of time in order to exclusively keep the center-most eighteen frames. Thereafter, the resulting eighteen SF vectors that constitute the smooth sampling matrix undergo a logarithmic transformation to adjust the SF selection to the human visual system’s sensitivity to SFs (log sampling matrix; Fig. 4f; see ref.^10^). As a result, a 256 × 18 elements log sampling matrix is obtained. Thereafter, each one of the eighteen 256 element vectors is rotated about its origin to create a two-dimensional isotropic SF filter for each image frame (2D filters; Fig. 4g). Then, the 2D filters are iteratively multiplied point-wise with the corresponding padded Fourier transformed image for each frame (Fig. 4h), and an inverse fast Fourier transformation is applied to each product (Fig. 4i). At each frame iteration, the central part of the filtered image (256 × 256 pixels) is cropped and sequentially integrated into a video which constitutes the final stimulus for a given trial (Fig. 4j). On each trial, these frames were displayed in succession at the same rate of about 16–17 ms – in line with all monitors’ vertical retrace rate of 60 Hz – adding up to a stimulus on screen appearance of 300 ms per trial (see Fig. 1 for a stimulus example).

### Procedure

The experiment comprised three phases. The first phase was a practice in which participants learned sixteen faces, including eight Western Caucasians (four males) and eight East Asians (four males). The second phase aimed at individually adjusting the difficulty level of the task such that all the participants reached an accuracy criterion of 51%. The third phase was the main task, used to measure the SF tuning of the participants. In all three phases, the task was the same: on each trial, following a 500 ms centred fixation cross, one of the sixteen learned faces was randomly presented at the center of the computer screen, and the participant was asked to identify it. The task was set up as a block design, each block featuring either one of the face ethnicities. The face ethnicity order was counterbalanced between subjects. More details about each phase are provided in the following paragraphs.

In the first phase, participants first familiarized themselves with eight faces of one ethnicity during a fixed period of eight minutes. Each face was associated with a keyboard key, and participants needed to learn this association in order to identify the faces during the experiment. The assigned keys covered one keyboard row so that participants could place both hands comfortably, and were consistent for all participants. After this first eight-minute familiarization period, participants completed one practice block by trying to identify the eight learned faces (unfiltered). On each trial of the practice phase, participants were instructed to first fixate the fixation cross that appeared in the middle of the screen for a duration of 500 ms. Then, a face was displayed until a response was given, and appropriate feedback was transmitted on the screen following each trial. Subsequently, a second eight-minute period of familiarization was provided to learn the eight faces of the other ethnicity and their associated keyboard keys. After this second familiarization period, participants then completed a practice block by trying to identify these eight new unfiltered faces. Practice blocks of 160 trials went on, while alternating between both face ethnicities, until participants reached an accuracy of at least 92% for two consecutive blocks of both Western Caucasian and East Asian faces. Once this criterion was met, they could advance to the second phase, which implemented the dynamic SF filtering technique.

The second phase aimed to find the number of bubbles necessary to maintain an accuracy rate around 51% (the same threshold value as in Tardif *et al*.^11^ was used for the purpose of replication). Adjusting the number of bubbles is a procedure typically applied when using the SF Bubbles method to keep participants’ performance level up to a fixed threshold. On one hand, this measure avoids dramatically hindering participants’ accuracy which, if not significantly above chance level, offsets the validity of correct trials. On the other hand, it prevents participants from reaching a high level performance plateau making it difficult to reveal which SF information is actually useful and which information is superfluous. In practice, increasing the number of bubbles means that, on average, more SF information will be visible to the participant, whereas decreasing it means that, on average, less SF information will be visible to the participant. More specifically, the number of bubbles corresponds to the number of target SFs sampled across one trial (i.e. the number of ‘ones’ distributed across the ‘raw sampling matrix’). Since the distribution of bubbles across the ‘raw sampling matrix’ is random, a higher number of bubbles can either increase the range of SF bands selected within one trial or, if the bubbles mostly fall within a small range of SFs, increase the magnitude (i.e. the intensity at which the SFs are present in the stimulus) of the selected SF bands. The procedure during that second phase was essentially the same as for the practice trials, but the faces were filtered using the transient SF Bubbles method explained above, and no feedback was given. Crucially, the stimulus was presented to the participant for 300 ms, and followed by a high contrast random noise mask. Participants completed blocks of 150 trials, alternating between blocks of Western Caucasian and East Asian faces in the same order as for the previous task. The number of bubbles was adjusted on a trial-by-trial basis using QUEST^77^. QUEST is a Bayesian adaptive procedure which, as implemented in the present task, estimated on each trial the most probable number of bubbles needed by a participant to reach the target accuracy criterion. Participants completed as many blocks as needed to reach a stable number of bubbles with both face ethnicities. Once stability was achieved, the final number of bubbles needed to steadily attain threshold performance for blocks featuring the other-race faces (relative to the participant’s ethnicity) was thereafter applied to stimuli of both ethnicities during the main task. We chose to base the final number of bubbles on the other-race faces because participants were generally worse with such faces, and we wanted to avoid a chance-level performance with them. We used the same number of bubbles for both face conditions to ensure that they were comparable in terms of the total amount of visual information they conveyed.

From that point on, the main task (i.e. third phase) started. The procedure during the main task was the same as for the second phase, except that the number of bubbles was fixed to the value obtained during the second phase. Participants completed 30 blocks, each consisting of 100 trials, again alternating between blocks of Western Caucasian and East Asian faces. Accuracy rate was monitored after each block to make sure that it did not reach an upper criterion of 75% with the same-race faces. If the accuracy rate was higher than 75%, a new block from the second phase (i.e. in which bubbles are adjusted with QUEST) was completed to readjust the number of bubbles. Only the blocks from the main task were included in the analyses, for a total of 3,000 trials.

### Classification image computation

The analysis method is essentially hinged on an association between the participants’ accuracy and the dynamic SF filter applied at each trial. More specifically, a linear combination is performed across all trials by appointing a positive weight to the raw sampling matrices (Fig. 4c) that yielded a correct response, and a negative weight to those that led to an incorrect response. The values of these weights are calculated by transforming accuracy values on each trial (i.e. ones and zeros) into *Z* scores using the average and standard deviation of the participant’s accuracies. The result of this linear combination procedure is called a classification image. In fact, the procedure to compute the classification images is similar to a multiple linear regression in which there are 2304 independent variables (i.e. 128 spatial frequencies × 18 frames), and the dependent variable is the accuracy. In the present case, the classification images represent how strongly the availability of each SF on each frame is associated with the participant’s accuracy. For each participant, two classification images, one for each face ethnicity, were produced. Each classification image was then smoothed by convolving it with a 2-dimensional Gaussian kernel subtending standard deviations of 2.5 cpi by 1.3 time frames. Then, as was done for the experimental stimuli, a logarithmic transformation was applied to the classification images. Lastly, the classification image values were converted into *Z* scores using an estimate of the mean and standard deviation under the assumption of the null hypothesis, derived from a permutation procedure applied to the data. Finally, for each face ethnicity separately, and each cultural group, the individual classification images were summed together and divided by the square root of the number of participants.

## Electronic supplementary material

Supplementary Information
