# Supplementary material for: Time Course of Cultural Differences in Spatial Frequency Use for Face Identification
Source: Sci Rep. 2018 Jan 29;8:1816. doi: 10.1038/s41598-018-19971-1 (PMC5788938; doi:10.1038/s41598-018-19971-1)
Supplement: Supplementary file 1 — Supplementary Information [file 41598_2018_19971_MOESM1_ESM.pdf]

Time Course of Cultural Differences in Spatial Frequency Use for Face Identification

(Supplementary Information file)

Amanda Estéphan[1,2], Daniel Fiset[1], Camille Saumure[1], Marie-Pier Plouffe-Demers[1], Ye Zhang[3,4], Dan Sun[3,4] & Caroline Blais[1,*]

[1] Département de psychoéducation et de psychologie, Université du Québec en Outaouais

[2] Département de psychologie, Université du Québec à Montréal

[3] Institute of Psychological Science, Hangzhou Normal University

[4] Zhejiang Key Laboratory for Research in Assessment of Cognitive Impairments

*Corresponding author information:

Caroline Blais, Ph.D.
Département de psychoéducation et de psychologie
Université du Québec en Outaouais
C.P. 1250, Succ. Hull
Gatineau, Qc
J8X 3X7
Phone: 819-595-3900 # 2551
Email: caroline.blais@uqo.ca

1. Ideal Observer analyses

One key advantage of the SF Bubbles method, compared to traditional high-pass, low-pass, or band-pass filtering techniques, is its high sensitivity to variations in information use. Although this characteristic might be clear in theory, it is surely advisable to ensure that this method is optimally implemented as to yield accurate results in practice. The method's accuracy at uncovering the precise SFs that carry the information potentially diagnostic for a given task was effectively verified as part of the study in which it was originally featured (see ref. 1). However, the analyses presented in that former study do not allow to characterize the modulation of the method's resolution as a function of the specific SF sampled. Moreover, in the present study, a temporal component was introduced to the SF Bubbles technique which in itself deserves testing. Indeed, the method is meant to be able to unravel potentially subtle and rapid temporal variations in SF use, and thus designed to be equipped with a very fine sampling rate. Hence, we used an Ideal Observer analysis to assess 1) the dynamic SF filtering method's sensitivity to a temporal change in the diagnostic SF information, and 2) the modulation of resolution as a function of the specific SF sampled.

1.1. Material and stimuli

The model was run on MATLAB with the Psychophysics Toolbox[2, 3]. A total of four sequences of vertical sine wave gratings were generated: (1) all low SF gratings, (2) first half low SF gratings, second half high SF gratings, (3) first half high SF gratings, second half low SF gratings, and (4) all high SF gratings. Each sequence comprised eighteen – which corresponds to the number of SF filter frames used in the main experiment – SF grating images of 256×256 pixels in size. The single gratings themselves covered about two thirds of their image canvas, had an invariable phase and a SF of either two cycles per image (cpi) –

for the "low" SF ones – or seventeen cpi – for the "high" SF ones. The stimuli were filtered using the transient SF filtering technique described in the main text.

1.2. Procedure

The Ideal Observer was programmed to select the matching sine wave grating sequence, among four choices (i.e. the four sequences listed above), to a target sequence. The target varied across trials and was filtered using our transient SF Bubbles method. White Gaussian noise was added and the signal-to-noise ratio of the target sequence was adjusted using QUEST[4] to maintain performance at 63% (midway between chance and perfect performance, rounded). The Ideal Observer performed a total of ten thousand trials. The number of bubbles was kept constant at a value of 600, which corresponds to the average number of bubbles needed by four pilot participants to reach the performance threshold in the face identification task described in the main text (two Canadians, two Chinese).

1.3. Analyses and results

Classification images were produced separately for each sequence of gratings using the procedure described in the *Methods* section of the main text. The Cluster test ($p < 0.05$; FWHM = 4.47; Zcrit= 3.0) from the Stat4CI toolbox[5] was used to measure the statistical significance of the classification images (see Supplementary Fig. S1). Following the application of the Cluster test, the SF tuning peaks for all significant clusters were calculated across frames using the 50% ASFM procedure described in the *Results* section of the main text.

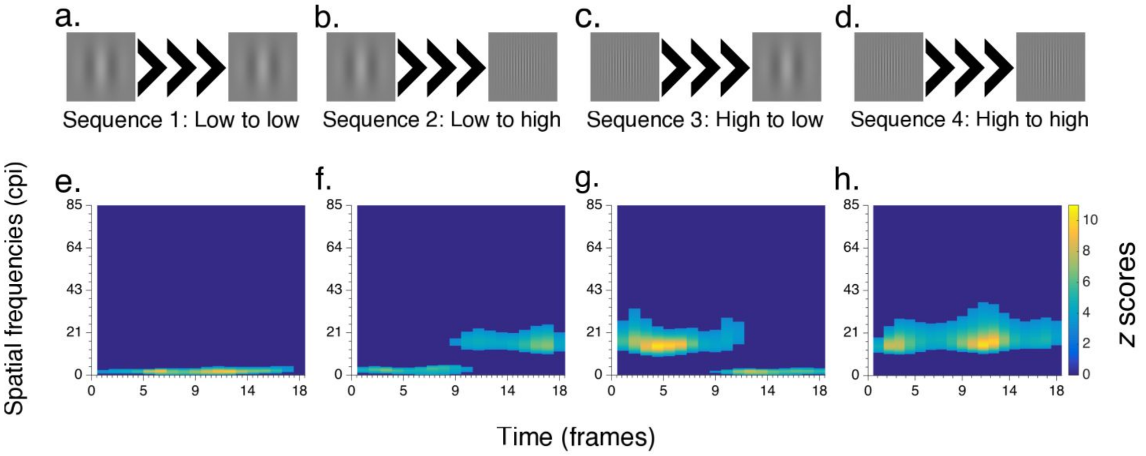

Figure S1. Illustration of the four possible target spatial frequency gabor sequences (a to d) and their corresponding classification images (e to h) representing the spatial frequency filters useful for correct sequence matching.

1.3.1. Method's sensitivity to a temporal change in the diagnostic SF information

Sequences 2 and 3 were the ones in which a change in the time course of the SF use should be observed. Accordingly, for "sequence 2" as target, a significant low SF cluster between 1.07 and 4.65 cpi – peaking at 3.12 cpi – spans across frames 1 to 10, and a high SF cluster between 14.16 and 22.16 – peaking at 16.75 cpi – spans across frames 9 to 18. Inversely, for "sequence 3" as target, a significant high SF cluster between 13.36 and 27.46 cpi – peaking at 18.15 cpi – spans across frames 1 to 11, and a low SF cluster between 0.73 and 3.00 – peaking at 1.89 cpi – spans across frames 9 to 18.

For both sequences, an overlap of low and high SFs is noticeable at frames 9 to 11. This overlap can be explained by the parameter selected for the gaussian smoothing across frames. In fact, as explained in the *Methods* section of the main text, the raw sampling matrix is convolved with a Gaussian kernel to avoid abrupt changes in the SFs sampled. The temporal dimension of the Gaussian kernel has a FWHM covering about three frames. This entails that when transitioning from low to high SFs (or vice versa) between frames 9 and 10,

at least half of the low (or high) SF information contained in the first half of the frame sequence (i.e. 1 to 9) is available in the two frames located at each side of the last frame of that first sequence half (i.e. 8 and 10), and at least half of the high (or low) SF information that makes up the second half of the frame sequence (i.e. 10 to 18) is also available in the two frames located at each side of the first frame from that second sequence half (i.e. 9 and 11). Although the observed overlap of SF subsets warrants careful interpretations of the results, this by-product of the technique is adequately revealed by the manipulation check, and highlights the suitable temporal resolution of the method.

1.3.2. Modulation of resolution as a function of the specific SF sampled

When “sequence 1” is presented as target, only low SFs, ranging from 0.75 to 3.78 cpi – with an average SF tuning peak of 2.02 cpi – across all frames, are significantly useful for the Ideal Observer to correctly discriminate the target from other sequences. For a target sequence composed solely of gratings with a SF of two cpi, SF filters that allow for information near two cpi to be available at high intensity, throughout all frames, are most optimal for the Ideal Observer. As for “sequence 4” as target, only high SFs ranging from 13.15 to 30.20 cpi – with an average SF tuning peak of 18.28 cpi – are significant across all frames. This sequence was exclusively made up of gratings with a SF of seventeen cpi, and the results indeed reveal the usefulness of SF filters through which information closest to seventeen cpi is available at high intensity across all frames.

Globally, the results obtained with the Ideal Observer confirm that our method offers an adequate spatial resolution, which can be especially observed for low SFs. It is nonetheless worth noting that the presence of wider range significant clusters for higher SFs in the classification images obtained in the main task stems from the application of a logarithmic SF

sampling technique which, as explained in the *Methods* section of the main text, takes into account the visual system's relative sensitivity to SFs. Hence, to examine in greater detail the relationship between the sampled SF and the spatial resolution of the filter, we conducted another analysis in which an Ideal Observer algorithm was required to find among 2 forced choices the matching sine wave grating to a target grating. Trials were performed using gratings of various SFs, while consistently combining two gratings five SFs apart. In the same way as the previous Ideal Observer analysis, the target was randomly selected at each trial and filtered with the SF sampling algorithm described in the *Methods* section of the main text. However, for this purpose the SF filters were created by convolving a simple one-dimensional Gaussian kernel – thus excluding the dimension of time – with a raw SF sampling vector, after which a logarithmic resampling of the resulting smooth sampling vector was conducted. The results of this analysis show that the spatial resolution of the SFs that appear to be significantly useful for the Ideal Observer to correctly match the grating becomes dramatically lower around 22 cpi (see Supplementary Fig. S2).

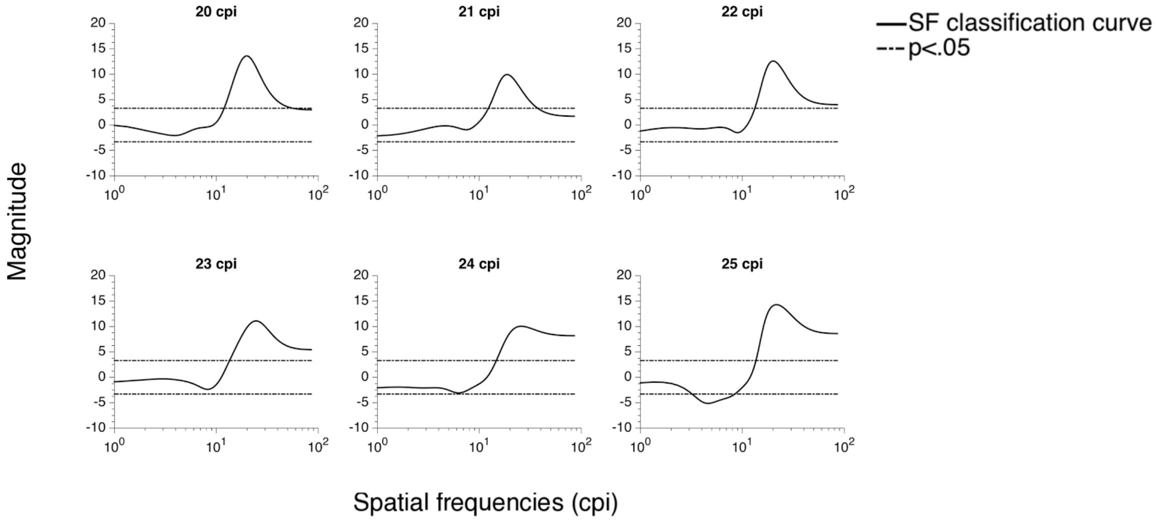

Figure S2. Illustration of the classification curves, representing the spatial frequency filters useful for correct grating matching, for spatial frequencies from 20 to 25.

2. Classification image analysis with participants' accuracy as factor (results)

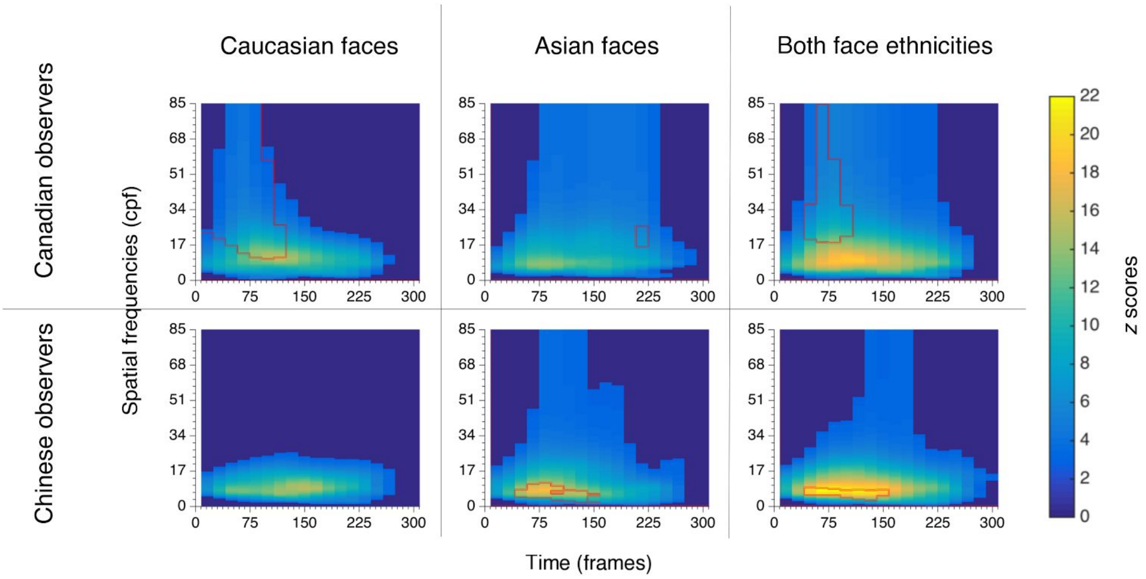

Figure S3. Classification images illustrating Canadian and Chinese observers' significant use of spatial frequencies across time, for Caucasian faces, Asian faces and both face ethnicities combined. Results obtained from subgroups of 12 Canadian and 12 Chinese participants, matched according to accuracy rate. Group differences (i.e. Canadian observers - Chinese observers) are marked for each group and stimulus category: red edges delineate significant SF use biases for each cultural group.

3. Bootstrap analysis of cultural differences in group classification images (results)

In order to simplify the description of the following cultural differences across time, the mean lower and higher SF bounds were derived for each SF × time cluster; however, the lower and higher bounds of the SF clusters on each frame separately are available on Fig. S4. The results that are shown here represent the cultural differences in SF utilization that have been revealed for 95% of our bootstrap subsamples. On the appended figure (i.e. Fig. S4), these results (delineated in red) were added over the original group classification images featured in the *Results* section of the main article.

For Canadian participants, a high SF cluster, ranging from 14.4 to 49.7 cpf, is shown as soon as the stimulus appears and for a steady 200 ms, for the identification of Western Caucasian faces (i.e. Fig. S4, first column); a high SF cluster appears much later and more briefly for East Asian faces (from 17.9 to 41.2 cpf, between 134 and 233 ms; i.e. Fig. S4, second column). When both face ethnicities are considered together (i.e. Fig. S4, last column), results show a high SF cluster between 15.7 and 56.3 cpf as early as 16.67 ms following stimulus onset and for the next 200 ms of stimulus presentation. Interestingly, for Canadian participants, low SF clusters, similar to the ones revealed in the *Results* section of our main article, also appear [i.e. from 3.4 to 7.1 cpf, between 184 and 267 ms, for Western Caucasian faces (see Fig. S4, first column); from 7.2 to 9.3 cpf, between 200 and 250 ms, for both faces ethnicities combined (see Fig. S4, last column)]. For East Asian faces, clusters of both 3.3 to 3.7 cpf and 8 to 10 cpf appear respectively during the first 16.67 ms and the last 33 ms (i.e. from 267 to 300 ms). However, this effect might be caused by the bootstrap method's high sensitivity.

For Chinese participants, a low SF cluster, between 3.3 and 4.8 cpf, starts at 83 ms and ends at 267 ms, with East Asian faces (i.e. Fig. S4, second column). However, with Western Caucasian faces (i.e. Fig. S4, first column), only a very small low SF cluster of 1.3 cpf appears during the first 16.67 ms, but this is likely again due to the high sensitivity of bootstrap method. Nonetheless, the absence of any important SF cluster with other-race faces for Chinese participants is consistent with the absence of a significant SF bias for those participants, with other-race faces, in our main results (see *Results* section of the main article). However, no SF cluster was revealed when both face ethnicities were combined (i.e. Fig. S4, last column), suggesting that the group differences found in this condition were less robust for Chinese participants.

If we reduce our confidence interval criterion to 90%, low SF clusters that resemble the ones initially found for Chinese participants in our main analysis start to appear for East Asian faces [1 to 2 cpf (during the first 16.67 ms), 6.7 to 8 cpf (around 33 ms), 5.7 to 6 cpf (around 100 ms), 0.3 cpf (between 150 and 183 ms)] as well as for both face ethnicities combined [0.3 to 1 cpf (during the first 16.67 ms), 6.2 to 7.2 cpf (between 33 and 67 ms), 4.7 to 5.3 cpf (between 100 and 133 ms), 0.3 to 0.7 cpf (between 167 and 200 ms)]. These clusters become even more prominent with an 85% confidence interval, for East Asian faces [0.7 to 2 cpf (during the first 16.67 ms), 6.2 to 8.2 cpf (between 33 and 67 ms), 5.3 to 6.7 cpf (between 83 and 117 ms), 0.3 cpf (between 133 and 200 ms)] and both face ethnicities combined [0.3 to 1.3 cpf (during the first 16.67 ms), 5.3 to 8.3 cpf (between 33 and 67 ms), 4 to 5.8 cpf (between 83 and 133 ms), 0.3 to 0.8 cpf (between 150 and 200 ms)].

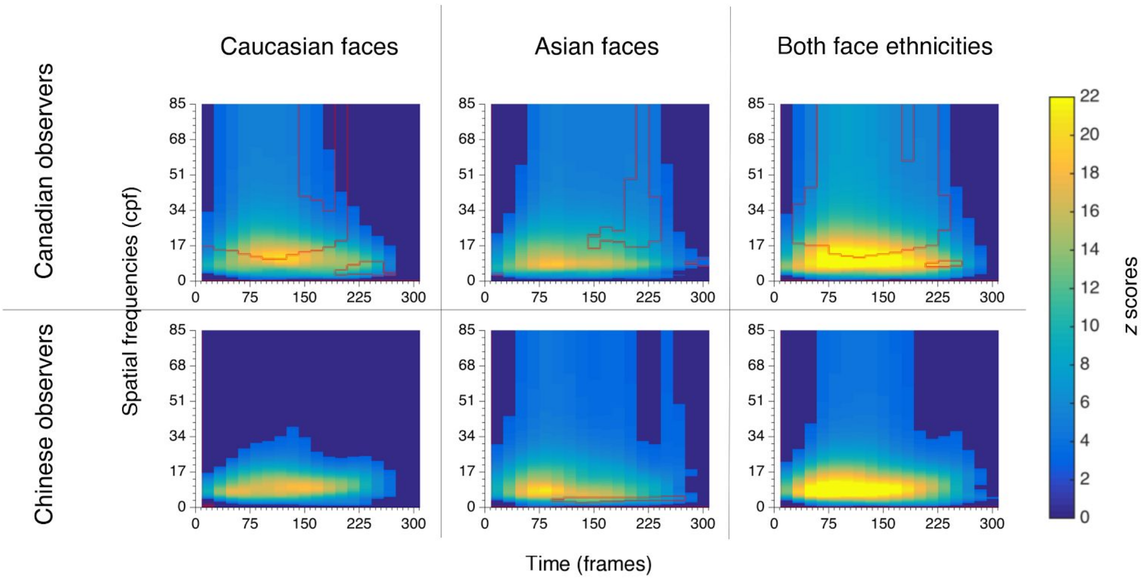

Figure S4. Classification images illustrating Canadian and Chinese observers' significant use of spatial frequencies across time, for Caucasian faces, Asian faces and both face ethnicities combined. Results obtained from 1000 bootstrap resamples. Group differences (i.e. Canadian observers - Chinese observers) are marked for each cultural group and stimulus category: red edges delineate cultural SF use biases that were revealed for 95% of subsamples.

4. References

1. Willenbockel, V. *et al.* Does face inversion change spatial frequency tuning? *Journal of Experimental Psychology: Human Perception and Performance* 36, 122–135 (2010a).

2. Brainard, D. H. The psychophysics toolbox. *Spatial vision* 10, 433–436 (1997).

3. Pelli, D. G. The videotoolbox software for visual psychophysics: Transforming numbers into movies. *Spatial vision* 10, 437–442 (1997).

4. Watson, A. B. & Pelli, D. G. Quest: A bayesian adaptive psychometric method. *Perception and psychophysics* 33, 113–120 (1983).

5. Chauvin, A., Worsley, K. J., Schyns, P. G., Arguin, M. & Gosselin, F. Accurate statistical tests for smooth classification images. *Journal of Vision* 5, 659–667 (2005).
